# Evaluation of the effective dose of amygdalin for the improvement of antioxidant gene expression and suppression of oxidative damage in mice

**DOI:** 10.7717/peerj.9232

**Published:** 2020-05-21

**Authors:** Sarah Albogami, Aziza Hassan, Nibal Ahmed, Alaa Alnefaie, Afnan Alattas, Lama Alquthami, Afaf Alharbi

**Affiliations:** 1 Department of Biotechnology, Faculty of Science, Taif University, Taif, Makkah, Kingdom of Saudi Arabia; 2 Department of Cell Biology, National Research Centre, Dokki, Cairo, Egypt; 3 Department of Biology, Faculty of Science, Taif University, Taif, Makkah, Kingdom of Saudi Arabia; 4 Department of Pathology, Animal Reproduction Research Institute, Cairo, Egypt; 5 General Department of Education, Taif, Makkah, Kingdom of Saudi Arabia

**Keywords:** Amygdalin, Hepatic and testicular tissues, GSH-Px, SODs mRNA expression, LPO, Histopathology, Antioxidant

## Abstract

**Background:**

Little is known regarding the toxic and therapeutic doses of amygdalin. Treatment regimens and schedules can vary between humans and animal models, and there have been reports of cyanide toxicity due to amygdalin use.

**Objective:**

The aim of this study was to evaluate the effect of different doses of amygdalin on antioxidant gene expression and suppression of oxidative damage in mice.

**Methods:**

Forty adult male mice were divided randomly into four groups (*n* = 10) as follows and treated orally for two weeks: a control group treated with saline solution, a group treated with amygdalin at 200 mg/kg body weight, a group treated with amygdalin at 100 mg/kg body weight, and a group treated with amygdalin at 50 mg/kg body weight. Liver and testis samples were collected for gene expression, biochemical and histopathological analyses.

**Results:**

The mice treated with medium-dose amygdalin (100 mg/kg) showed upregulated mRNA expression of glutathione peroxidase (*P* < 0.01) and superoxide dismutase (*P* < 0.05) and significantly decreased lipid peroxidation (*P* < 0.05) in hepatic and testicular tissues compared to those in the untreated groups (controls), with mild histopathological effects. The mice treated with high-dose of amygdalin (200 mg/kg) showed downregulated mRNA expression of glutathione peroxidase and superoxide dismutase (*P* < 0.01) and significantly increased lipid peroxidation (*P* < 0.05) in both hepatic and testicular tissues compared to those in the untreated groups (controls), with an apparent effect at the histopathological level. No effects were observed in the mice treated with low-dose amygdalin (50 mg/kg) at the gene, protein and histopathological level.

**Conclusion:**

Low-and medium-dose amygdalin did not induce toxicity in the hepatic and testicular tissues of male mice, unlike high-dose amygdalin, which had a negative effect on oxidative balance in mice. Therefore, amygdalin at a moderate dose may improve oxidative balance in mice.

## Introduction

The aromatic cyanogenic compound amygdalin (C_20_H_27_NO_11_) occurs naturally in the seeds of apples, pits of apricots and peaches, and bitter almonds (Jaswal, Palanivelu & Ramalingam, 2018; Li, Swain & Poulton, 1992; London-Shafir, Shafir & Eisikowitch, 2003; Luo et al., 2018). It is a bioactive compound consisting of glucose, hydrocyanic acid (an antineoplastic compound), and benzaldehyde that exerts an analgesic effect (Hwang et al., 2008a). This compound has long been utilized for its unique medicinal properties, as it has been shown to exert anticancer effects (Saleem et al., 2018).

Amygdalin is nontoxic; however, once it digested by certain enzymes such as β-D-glucosidase, which is commonly found in the small intestine of humans and in various commonly consumed foods (Strugala et al., 1995), it leads to the production of toxic hydrocyanic acid (Haisman & Knight, 1967; Holzbecher, Moss & Ellenberger, 1984). Hydrocyanic acid and amygdalin have been shown to exert anti-asthmatic and antitussive effects, stimulate the respiratory center, and potentially affect the digestive system (Badr & Tawfik, 2010).

Other studies have shown that amygdalin exerts additional pharmacological effects, including prevention of pulmonary fibrosis, inhibition of renal interstitial fibrosis, suppression and regulation of the immune system, and resistance to hyperoxia-induced lung injury, as well as anti-inflammatory, antitumor and anti-ulcer activities (Chang et al., 2005; Guo et al., 2013; Hwang et al., 2008b; Yan et al., 2006; Zhu et al., 2004). Amygdalin has been used to treat leprosy, asthma, emphysema, bronchitis, vitiligo and colorectal cancer (Chang et al., 2005; Do et al., 2006; Song & Xu, 2014). The antitumor effect of amygdalin occurs following its decomposition into hydrocyanic acid, whereas its analgesic effect occurs following its decomposition into benzaldehyde (Davignon, Trissel & Kleinman, 1978; Fukuda et al., 2003; Moertel et al., 1982; Song & Xu, 2014).

Although some studies have indicated that amygdalin has anticancer effects, other studies have suggested the opposite and that it can cause potentially fatal cyanide poisoning (Blaheta et al., 2016; Humbert, Tress & Braico, 1977; Khandekar & Edelman, 1979; Lee & Moon, 2016; Milazzo, Horneber & Ernst, 2015; Milazzo, Lejeune & Ernst, 2007; Newton et al., 1981; Zhou et al., 2012). In humans, amygdalin ingestion may lead to cyanide poisoning-like symptoms, including headache, nausea, dizziness, vomiting, liver damage, nerve damage, hypotension, coma and, in extreme cases, death (Chang et al., 2006). Currently, limited information is available on the in vivo and in vitro antioxidant activities of amygdalin (Durmaz & Alpaslan, 2007). Clinical studies have provided insufficient evidence to aid its approval by the Food and Drug Administration as a cancer treatment, and the potential toxicity associated with its use has not been revealed (Bitting, 1978). However, despite the lack of approval and absence of positive clinical trials investigating the anticancer effects of amygdalin, this compound continues to be manufactured and used as an anticancer therapy at alternative (holistic) cancer treatment clinics found in some parts of Europe and Mexico (Halenar et al., 2015).

Little is known regarding the toxic and therapeutic doses of amygdalin. Treatment regimens and schedules can vary between humans and animal models, and there have been reports of cyanide toxicity due to amygdalin use.

Therefore, in the present study, we aimed to elucidate the effect of high, medium and low doses of amygdalin on antioxidant gene expression and enzyme activity in the testicular and hepatic tissues of male mice. We also assessed histopathological alterations in these tissues following amygdalin administration. The findings of our study may enhance our understanding of the toxicity and efficacy of amygdalin to advance its development as an anticancer treatment.

## Materials and Methods

### Amygdalin

Amygdalin of purity ≥98% was purchased from LKT Laboratories, Inc. (St. Paul, MN, USA). It was freshly prepared diluted doses of 200, 100 and 50 mg/kg of body weight by dissolving the stock powder in (0.2 mL) saline solution. The control group received saline solution (0.2 mL) (Moslehi et al., 2018). The dose range of amygdalin used in this study was chosen based on the fact that lethal oral doses (LD50) for cyanide is between 2.13 and 6 mg kg^−1^ b.w., and it has been confirmed that 59 mg of cyanide is released from 1 g of amygdalin (Chain EPoCitF, 2016; FAO/WHO, 2012). The tested does and duration of the experiment were determined according to previous reports (Alexander et al., 2016; Blaheta et al., 2016; Halenar et al., 2017; Kolesár et al., 2015; Liu et al., 2017; Qadir & Fatima, 2017; Song & Xu, 2014).

### Experimental animals

Forty adult male albino mice (*Mus musculus*; average weight, 20–25 g), which were chosen to avoid data interference from hormonal variations, were purchased in November 2018 from King Fahd Center for Medical Research (King Abdulaziz University, Jeddah, Saudi Arabia). The mice were kept in a room maintained at a temperature of 25 ± 2 °C with a 12-h light/dark cycle, and they had free access to food and water. This study was accomplished in strict compliance with standard recommendations in the Guide for the Care and Use of Laboratory Animals of the National Institutes of Health (National Research Council Committee for the Update of the Guide for the C, & Use of Laboratory A, 2011), and Animal Research: Reporting of In Vivo Experiments guidelines (Kilkenny et al., 2010). The protocol was approved by the Committee on the Ethics of Animal Experiments of Taif University, Taif, KSA (439-6093). The mice were sacrificed under ketamine/xylazine anesthesia; efforts were made to reduce stress and pain to the mice.

### Experimental design

**Figure 1:**
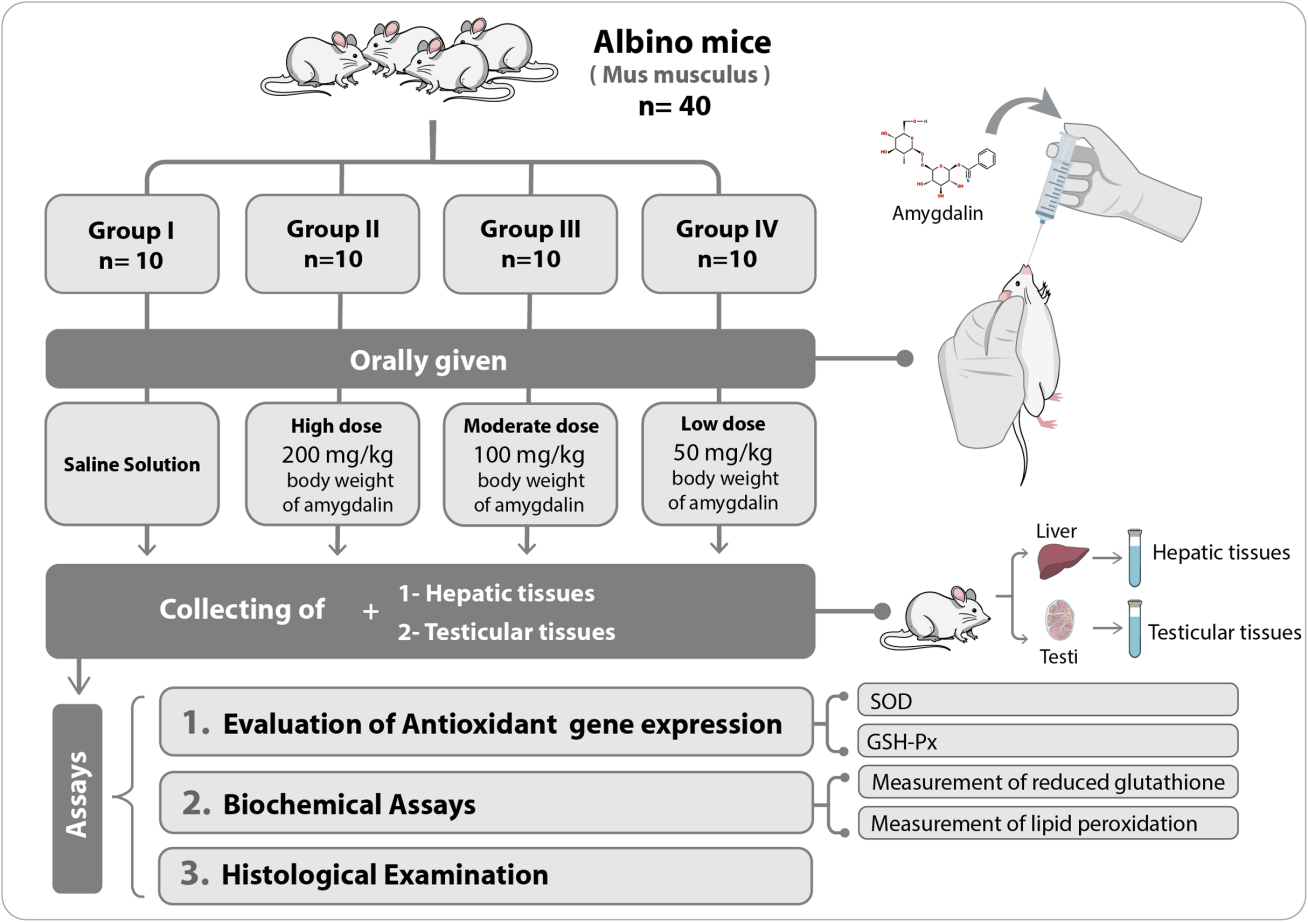
Schematic representation of the experimental design. SOD, superoxide dismutase; GSH-Px, glutathione peroxidase.

The mice were first allowed to adapt to their new habitat for seven days. Thereafter, they were divided into four experimental groups (*n* = 10 each) and were orally administered the following treatments once per day for two weeks: Group I (Control), received saline solution (0.2 mL) (*n* = 10); Group II, received high-dose (200 mg/kg body weight) amygdalin solution; Group III, received medium-dose (100 mg/kg body weight) amygdalin solution; and Group IV, received low-dose (50 mg/kg body weight) amygdalin solution. At the end of the 2-week treatment period, the mice were euthanized, and hepatic and testicular tissues were collected and rinsed with cold buffered saline. For histopathological examination, a small portion from each tissue was stored in 5% glutaraldehyde. The remaining tissue samples were stored at −80 °C until use for RNA isolation and biochemical assays. Figure 1 shows a summary of the experimental design.

### Biochemical assays

#### Preparation of tissue homogenates for biochemical assays

Hepatic and testicular tissues were homogenized separately in a homogenizer using ice-cold homogenizing medium (0.1 M sodium phosphate and five mM EDTA, pH 8) at a tissue concentration of 25 mg/mL. The homogenates were centrifuged for 20 min at 12,000×*g*, and the supernatants were transferred into new test tubes and used in the subsequent biochemical assays (Yao et al., 2003).

#### Measurement of lipid peroxidation activity

Lipid peroxide level was determined using thiobarbituric acid of ≥99% purity (Sigma Chemical Co., St. Louis, MO, USA), as described previously (Ohkawa, Ohishi & Yagi, 1979). Briefly, 100 µL of each hepatic and testicular tissue homogenate was mixed with 1.5 mL of 20% glacial acetic acid, 1.5 mL of 0.8% thiobarbituric acid, 200 µL of 8.1% SDS and 600 µL of distilled water. The mixture was vortexed and heated at 95 °C for 1 h in a water bath. Thereafter, the test tubes were cooled to 25 °C and centrifuged for 10 min at 10,000×*g*. The absorbance of each sample was measured at 532 nm using a UV-visible spectrophotometer (PerkinElmer, Waltham, MA, USA). Six replicates were measured per sample and the final concentrations are presented as nmol/g tissue.

#### Measurement of reduced glutathione activity

The level of reduced measurement of reduced glutathione (GSH) in the liver and testis was determined using a method described previously (Ellman, 1959). Briefly, 200 µL of hepatic and testicular tissue homogenate was mixed with 400 µL of lysis buffer (10 mM Tris, 0.2% Triton X-100, and 1 mM EDTA). The mixture was incubated on ice for 30 min and centrifuged for 10 min at 10,000×*g*. Two hundred microliters of the supernatant was transferred into a new test tube and mixed with 400 µL of 0.1 M sodium phosphate (pH 8.0) containing one mM EDTA and 100 µL of 10 µM 5,5′-Dithiobis(2-nitrobenzoic acid) (DTNB; Ellman’s Reagent). The absorbance of each sample was measured at 412 nm against a blank using a UV-visible spectrophotometer. Six technical replicates were measured per sample and the GSH level (expressed as mmol/g of sample) was calculated by interpolation on a standard curve constructed using known concentrations of GSH.

### Evaluation of gene expression

#### RNA isolation

Small sections of the liver and testis tissues were homogenized in one mL of TRIzol reagent (Invitrogen, Carlsbad, CA, USA) and the total RNA was isolated in accordance with the manufacturer’s instructions. The isolated RNA was dissolved in diethyl pyrocarbonate (DEPC)-treated water, and then stored at −80 °C until use. The purity and yield of the isolated RNA were evaluated using the NanoDrop^®^ ND-1000 UV-Vis Spectrophotometer (Thermo Fisher, Waltham, MA, USA).

#### Reverse transcription-polymerase chain reaction

Complementary DNA (cDNA) was produced from the isolated RNA using the Access Reverse transcription-polymerase chain reaction (RT-PCR) System (Promega Corporation, Madison, WI, USA) according to the manufacturer’s instructions. The PCR mixture contained one mM antisense and sense primers (Table 1).

**Table 1:** Primer sequences for each antioxidant gene.

Gene	Primer	Sequence 5′–3′	Length (bp)	Tm	GC%	Product size (bp)
GSH-Px	F	GGGGAAGCCAGTCCTTCATT	20	59.67	55.00	403
	R	AAAGGCAGGGAAGTAACGGT	20	59.23	50.00	
SOD	F	AGGGAACCATCCACTTCGAG	20	59.09	55.00	415
	R	TGCGCAATCCCAATCACTCC	20	59.03	55.00	
GAPDH	F	GGTGCTGAGTATGTCGTGGA	20	59.47	55.00	449
	R	ACATTGGGGGTAGGAACACG	20	59.67	55.00	

**Note:**

Forward (F) and reverse (R) primer sequences for GSH-Px, SOD, and GAPDH (internal standard) with the expected PCR product size. GAPDH, glyceraldehyde-3-phosphate dehydrogenase.

First-strand cDNA was synthesized under the following conditions: 45 °C for 45 min (1 cycle) for reverse transcription, followed by 94 °C for 2 min (1 cycle) for avian myeloblastosis virus reverse transcriptase inactivation and RNA/cDNA/primer denaturation. The following conditions were used for the second-strand cDNA synthesis and PCR amplification: 94 °C for 30 s for denaturation; 60 °C for 1 min for annealing; 68 °C for 2 min for extension, 40 cycles; and 68 °C for 7 min for final extension, 1 cycle.

#### Electrophoresis

The RT-PCR products were separated on 1% agarose gels, with a 100-bp DNA marker as a molecular standard (Bioland Scientific LLC, CA, USA), and then visualized using the Accuris E3000 UV Transilluminator (Benchmark Scientific, Edison, NJ, USA). The gel images were analyzed using Gel-Pro (version 3.1, Silk Scientific, Inc., Orem, UT, USA).

#### Histopathological examination

The mice from the control and treated groups were dissected to remove the liver and testes. The specimens were immediately immersed in 5% glutaraldehyde. After fixation, the specimens were trimmed, washed and dehydrated in ascending concentrations of ethyl alcohol, and then cleared in xylol. Finally, the samples were embedded in paraffin for 1.5 h at 50 °C in an oven. Serial thin sections (~4–6 µm thick) were cut and stained with hematoxylin and eosin (H&E) for general microscopic examination. Staining was carried out according to the method of Bancroft and Gamble (Bancroft & Gamble, 2008).

### Statistical analysis

One-way and two-way analysis of variance (ANOVA) was used for data analysis and Waller–Duncan post-hoc test was subsequently used to derive statistical significance between groups (Waller & Duncan, 1969). Data were expressed as mean ± standard deviation (SD), and differences were considered significant at *P* < 0.05. Compute correlation between Amygdalin concentrations vs. GSH-Px (mg/g) (in hepatic and testicular tissues) and Amygdalin concentrations vs. LPO (nmol/g) (in hepatic and testicular tissues) was performed by correlation XY analyses and *P* value calculated as two-tailed.

## Results

### Effect of amygdalin on GSH-Px and SOD mRNA expression

**Figure 2:**
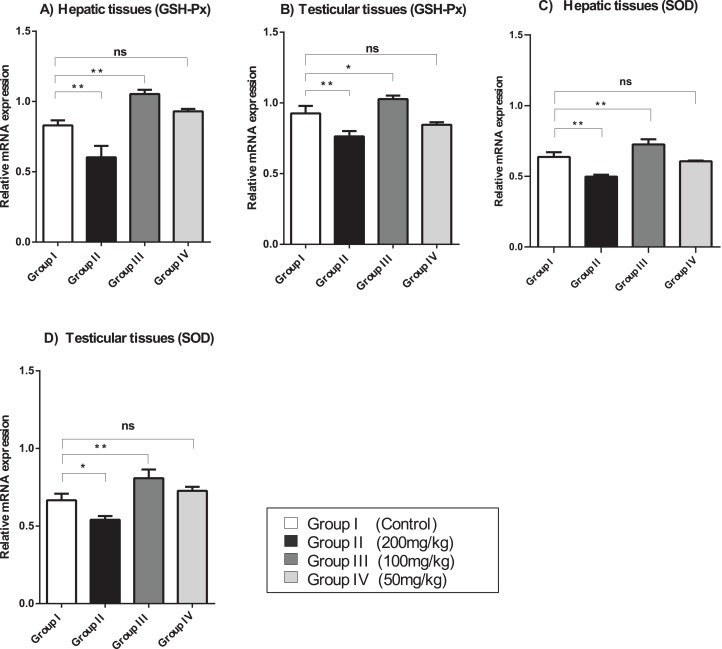
SOD, superoxide dismutase; GSH-Px, glutathione peroxidase. (A) Hepatic tissue GSH-Px. (B) Testicular tissue GSH-Px. (C) Hepatic tissue SOD. (D) Testicular tissue SOD. ANOVA was used for data analysis. **P < 0.01; *P < 0.05; ns, not significant. The values are presented as mean ± SD (*n* = 3).

The relative mRNA expression levels of GSH-Px and SOD in the hepatic and testicular tissues were evaluated using semi-quantitative (sq)-PCR analysis. As shown in Fig. 2, the mice treated with a relatively high dose (200 mg/kg) of amygdalin showed a significant decrease (*P* < 0.01) in GSH-Px and SOD gene expression levels in the hepatic and testicular tissues, compared with that in the control. The mice treated with 100 mg/kg of amygdalin showed significantly increased GSH-Px and SOD gene expression in both hepatic and testicular tissues (*P* < 0.01 and *P* < 0.05, respectively), compared with that in the control. Treatment with the low dose of amygdalin (50 mg/kg) did not significantly alter the gene expression of neither the hepatic nor testicular tissues relative to that in the control. These results show that high-dose amygdalin downregulated GSH-Px and SOD mRNA expression, whereas the medium dose upregulated their mRNA expression and the low dose exerted no effect on the expression of these genes.

### Effect of amygdalin on oxidative stress parameters

#### Measurement of GSH activity

**Figure 3:**
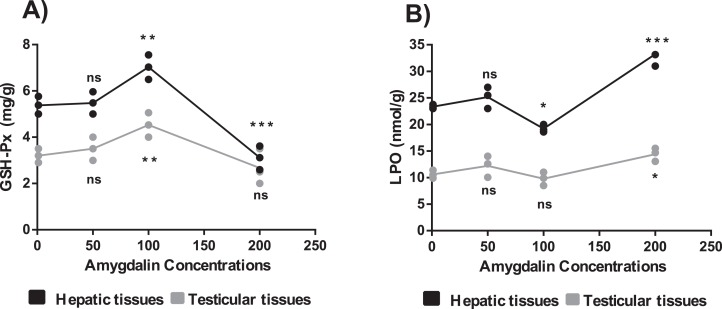
Effect of different doses of amygdalin on glutathione peroxidase (GSH-Px) and lipid peroxidation (LPO) activities in the hepatic and testicular tissues of mice. (A) GSH-Px (mg/g) activities. (B) LPO activities (nmol/g). black: Hepatic tissue, Grey: Testicular tissues. Two-way ANOVA was used for data analysis. **P < 0.01; *P < 0.05; ns, not significant. The values are presented as mean ± SD (*n* = 3).

The mice treated with high-dose amygdalin (200 mg/kg) presented a significant decrease in the GSH-Px activity level in the hepatic and testicular tissues (*P* < 0.05) compared with that in the control. The GSH-Px activity level in both hepatic and testicular tissues significantly increased (*P* < 0.05) after treatment with medium-dose (100 mg/kg) amygdalin compared with that in the control. The mice treated with low-dose (50 mg/kg) amygdalin did not show any significant differences in their GSH-Px activity levels compared with that in the control mice (Fig. 3A).

#### Measurement of LPO activity

The mice treated with high-dose amygdalin (200 mg/kg) (Group II) presented a significant increase in the LPO activity level (*P* < 0.05) in both hepatic and testicular tissues compared with that in the control group (Group I). The group treated with medium-dose amygdalin (100 mg/kg) (Group III) showed a statistically significant decrease in the LPO activity level (*P* < 0.05) in hepatic tissue but not in testicular tissues relative to that of the control group. The mice treated with low-dose (50 mg/kg) amygdalin did not show significant differences in their LPO activity level compared with that of the control group (Fig. 3B).

### Histological findings

#### Effects of amygdalin treatment on the histology of testicular tissue

**Figure 4:**
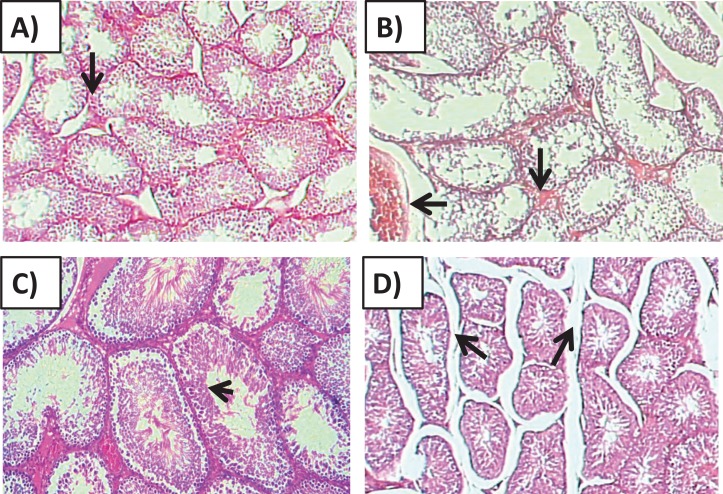
Effects of amygdalin on the histology of the testicular tissue of mice. (A) Testicular tissue of Group I (control) showing normal seminiferous tubules and interstitial tissues (H&E, ×100). (B) Testicular tissue of Group II (200 mg/kg) showing marked vascular congestion (black arrow), tubular vacuolar degeneration (black arrow), necrosis of the spermatogonia that line the seminiferous tubules, and interstitial edema (H&E, ×100). (C) Testicular tissue of group III (100 mg/kg) showing maintenance of normal seminiferous tubules with no histopathological alterations and complete spermatogenesis (arrow) (H&E, ×100). (D) Testicular tissue of Group IV (50 mg/kg) showing active spermatogenesis and normal cell arrangement in the lumen of the seminiferous tubules, with mild congestion (arrow) (H&E, ×40).

Histology of the testicular tissue of Group I (control)/(Fig. 4A) showed normal features with a thick fibrous capsule of the tunica albuginea. The basal lamina formed by myoid cells was found to line the seminiferous tubules. The histological results showed that all tubules contained their own epithelial cells, including Sertoli cells and germ cells at different stages, throughout spermatogenesis. The Sertoli cells had well-defined cytoplasm and typical, irregular nuclei.

**Figure 5:**
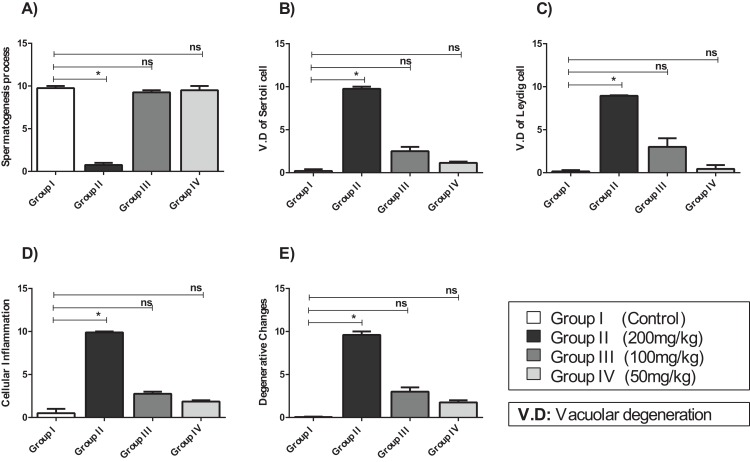
The histopathological alteration in the testicular tissue of mice. (A) Spermatogenesis process; (B) V.D of Sertoli cell; (C) V.D of Leydig cell; (D) cellular inflammation; (E) degenerative changes. One-way ANOVA was used for data analysis. *P < 0.05; ns, not significant. The values are presented as mean ± SD (*n* = 10).

Compared with that of the control group, the testicular tissue of Group II (200 mg/kg)/(Fig. 4B). Showed pathological alterations, including congestion of the interstitial blood vessels, necrosis and inflammation. The tubular lumen contained luminal cellular debris and only a few scattered clusters of spermatozoa. There was significant apparent vacuolar degeneration of Sertoli and spermatogenic cells, and necrosis of Leydig cells with rounded nuclei and deep eosinophilic cytoplasms (*P* < 0.05) (Figs. 5A–5E).

Compared with those of Group II, the testicular tissue of Group III (100 mg/kg)/ (Figs. 4C and 5) showed improved histological features, almost similar to those of the control group. There was evidence of normal interstitial cells and Leydig cells, and the epithelium of the seminiferous tubules showed distinct nuclei. Mature sperm bundles and a normal basement membrane were observed in the lumen.

The histopathology of the testicular tissue of Group IV (50 mg/kg)/(Figs. 4D and 5) revealed very similar features to that of the control group, with unaffected spermatogenesis at different stages of differentiation and maturation.

#### Effect of amygdalin on the histology of the hepatic tissue

**Figure 6:**
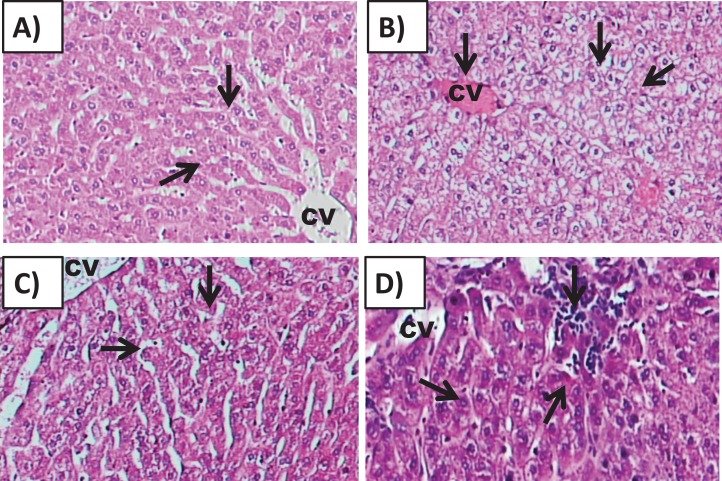
Effects of amygdalin on the histology of the hepatic tissue of mice. (A) Hepatic tissue of Group I (control) showing normal architecture, with normal hepatocytes in the hepatic cords (black arrows) and central vein (CV) (H&E, ×100). (B) Hepatic tissue of Group II (200 mg/kg) showing vascular congestion (black arrow), Kupffer cell activation, marked cytoplasmic vacuolization, and binucleated hepatocytes (black arrow) (H&E, ×100). (C) Hepatic tissue of Group III (100 mg/kg) showing normal hepatocytes and central vein (H&E, ×100). (D) Hepatic tissue of Group IV (50 mg/kg) showing normal histological features with mild inflammatory cell infiltration (red arrow) (H&E, ×100).

**Figure 7:**
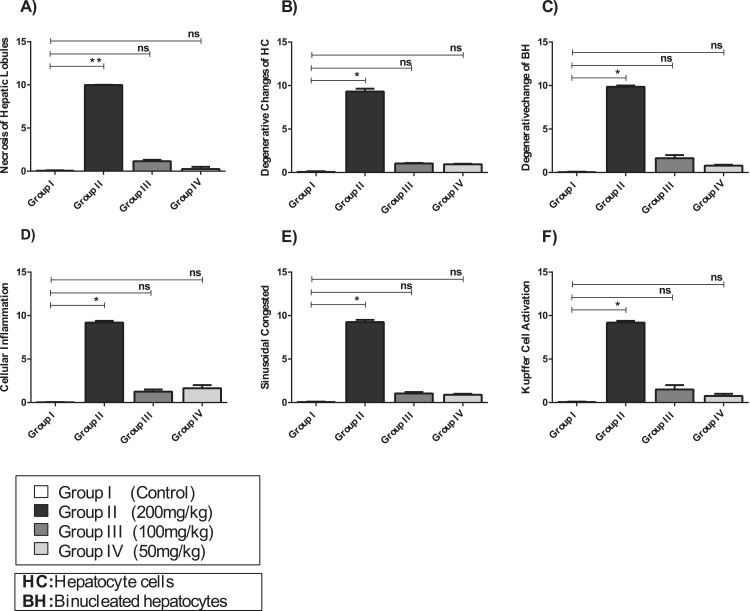
The histopathological alteration in the hepatic tissue of mice. (A) Necrosis of hepatic lobules; (B) degenerative changes of HC; (C) degenerative change of BH; (D) cellular inflammation; (E) sinusoidal congested; (F) kupffer cell activation. One-way ANOVA was used for data analysis. *P < 0.05; ns, not significant. The values are presented as mean ± SD (*n* = 10).

Examination of Group I (control)/(Fig. 6A) hepatic tissue revealed normal histoarchitecture of the intact portal areas and normal hepatic cells. Examination of Group II (200 mg/kg)/(Fig. 6B) hepatic tissue showed widespread necrosis and vacuolar degeneration throughout the hepatic lobules, which were especially evident in centrilobular cells (Fig. 7B). There was significant increase in severe congestion in the central veins and sinusoidal spaces, with areas of hemorrhage and inflammatory cell infiltration found almost adjacent to the portal areas, mainly by lymphocytes, plasma cells and macrophages. We also identified Kupffer cell activation, evident cytoplasmic vacuolization, and hepatocyte binucleation Within the liver capsule, inflammatory cell infiltration was linked to subcapsular congestion and edema (Figs. 7A, 7B, 7C, 7D, 7E and 7F).

The hepatic tissue of Group III (100 mg/kg)/(Figs. 6C and 7) showed a normal structure with central veins, peripheral altriad and hexagonal lobules embedded in the connective tissue. The hepatic tissue of group IV (50 mg/kg)/(Figs. 6D and 7) showed a normal appearance, within histological limits, with mild inflammatory cell infiltration, pathological alterations, or necrosis.

## Discussion

In the present study, the results of mRNA expression and biochemical analyses revealed that the mice treated with a relatively high dose of amygdalin (200 mg/kg) presented a significant decrease in GSH-Px and SOD expression in the hepatic tissue; however, this decrease was not significant in the testicular tissue. The biochemical analysis showed that the protein activity of GSH-Px in the hepatic and testicular tissues significantly decreased compared with that in the control group. In addition, there was a significant increase in the LPO level in the hepatic (33.160 ± 1.337 nmol/g; *P* < 0.05) and testicular tissues (13.043 ± 0.134 nmol/g; *P* < 0.05) compared with that in the control group.

The toxicity and efficacy of amygdalin in animal models have not been fully investigated, however it has been reported that orally administered amygdalin releases a high amount of cyanide (Mani et al., 2019). Amygdalin can be converted into hydrocyanic acid via the action of emulsion complex enzymes (Zhou et al., 2012) that contain β-D-glucosidase, which is found in foods, and microflora of the colon and small intestine (Blaheta et al., 2016); amygdalin can also undergo hydrolysis without enzymatic catalysis (Holzbecher, Moss & Ellenberger, 1984; Strugala et al., 1995), also it can be hydrolyzed by water into glucose, benzaldehyde, and hydrocyanic acid; however, boiling water causes amygdalin to epimerize (Savic et al., 2015). Cyanide, a potent neurotoxin, leads to the generation of reactive oxygen species (ROS), which may damage vital organs including the kidney, liver, brain and heart. Furthermore, oxidative stress occurs if the effects of free radicals were not alleviated (Kadiri & Asagba, 2019). Primarily, the toxic property of cyanide is attributed to its association with the cytochrome oxidase terminal in the respiratory pathway of the mitochondria creating a hinderance in the cells’ ability to use oxygen (Opyd et al., 2017). In humans, cyanide has been shown to cause highly acute toxicity, causing increased LPO levels and oxidative stress (Holzbecher, Moss & Ellenberger, 1984; Kamendulis et al., 2002). Mitochondrial dysfunction and increased metabolism in cancer cells, compared with those in normal cells, are the main causes of high levels of ROS. This mechanism may be associated with the anticancer effects of amygdalin by triggering several ROS-induced cell death pathways of cancer cells (Galadari et al., 2017).

A recent study reported that amygdalin causes toxicity after oral consumption but not intravenous administration; however, its mode of action and toxic doses have not been confirmed. Various oral doses of amygdalin have been shown to cause toxicity, which can be attributed to an eclectic gut consortium (Jaswal, Palanivelu & Ramalingam, 2018).

Studies have shown that different oxidants can deplete GSH-Px level due to oxidative stress in rats (El-Beshbishy et al., 2011). ROS-induced damage has been suggested to act via LPO in biological systems; however, the data obtained in this study showed that ROS overproduction due to high doses of amygdalin led to benzaldehyde overproduction. This may be due to Dakin oxidation, which is an organic redox reaction where ketones or benzaldehydes react with hydrogen peroxide to form carboxylate and benzenediol (Dakin, 1906). This process (i.e., oxidation of the carbonyl group) may play a key role in the oxidization of protein, including the production of protein carbonyls. Therefore, we hypothesize that the ROS produced due to amygdalin, and the subsequent benzaldehyde overproduction, may trigger protein oxidation before the LPO process.

Previous studies have shown that there is a close association between the liver and testis damage and increased GSH depletion and LPO caused by ROS with decreased levels of antioxidant enzymes (Aitken & Roman, 2008; Sies, 1997). The histopathological analysis of selective tissues showed severe degenerative changes in the liver of mice treated with high-dose amygdalin, compared with that of the control group and the other amygdalin-treated groups, which showed normal morphological patterns. In the present study, damage to the liver was characterized by vascular congestion, Kupffer cell activation, and marked cytoplasmic vacuolization. Similar histopathological injury was observed in the liver of rabbits and rats treated orally with cyanide (Avais et al., 2018; Okolie & Osagie, 1999; Sousa et al., 2002); however, exposure to cyanide compounds caused minimal hepatic degenerative changes in goats (Soto-Blanco, Stegelmeier & Górniak, 2005; Soto-Blanco et al., 2008), which confirm the results of the present study. Oral treatment with 200 mg/kg amygdalin caused pathological alterations in the testicular tissues, including necrosis, and an obvious decline in spermatogonia cell number. This caused epithelial disarray and loss of some of the internal spermatogonia layers in the basement membrane, resulting in cessation of spermatogenesis. We also observed vacuolar degeneration of Sertoli and spermatogenic cells and necrosis of Leydig cells showing deep eosinophilic cytoplasmic staining. This is similar to the results of the study of Shivanoor and David, who showed that exposure to cyanide caused oxidative damage in sperm biomolecules and reproductive organs, resulting in altered sperm function and toxicity to male genital and reproductive organs (Shivanoor & David, 2015).

Based on the results of this study, whenever the concentration of hydrogen cyanide and benzaldehyde is increased to a critical level, amygdalin can act as a pro-oxidant, altering the oxidative balance instead of ROS overproduction. Thereafter, the Dakin reaction may predominate, leading to protein oxidation instead of LPO, potentially explaining the changes caused by oxidative damage (Yigit, Yigit & Mavi, 2009). Treatment with medium-dose amygdalin (100 mg/kg) significantly modulated the mRNA expression of GSH-Px and SOD. Furthermore, the GSH-Px protein levels in both hepatic and testicular tissues were significantly improved compared with that in the control group. Moreover, compared with that in the control, the LPO level significantly decreased in the liver and testis. The effect of medium-dose amygdalin on the total GSH-Px level may be due to a direct antioxidant effect, which would upregulate the biosynthesis of GSH. This is consistent with the findings of previous studies, where some antioxidants can affect the levels of cellular GSH by direct antioxidant effects, resulting in increased biosynthesis of GSH or increased levels of other antioxidants, including vitamins E and A (Ochiai et al., 2004a, 2004b).

**Figure 8:**
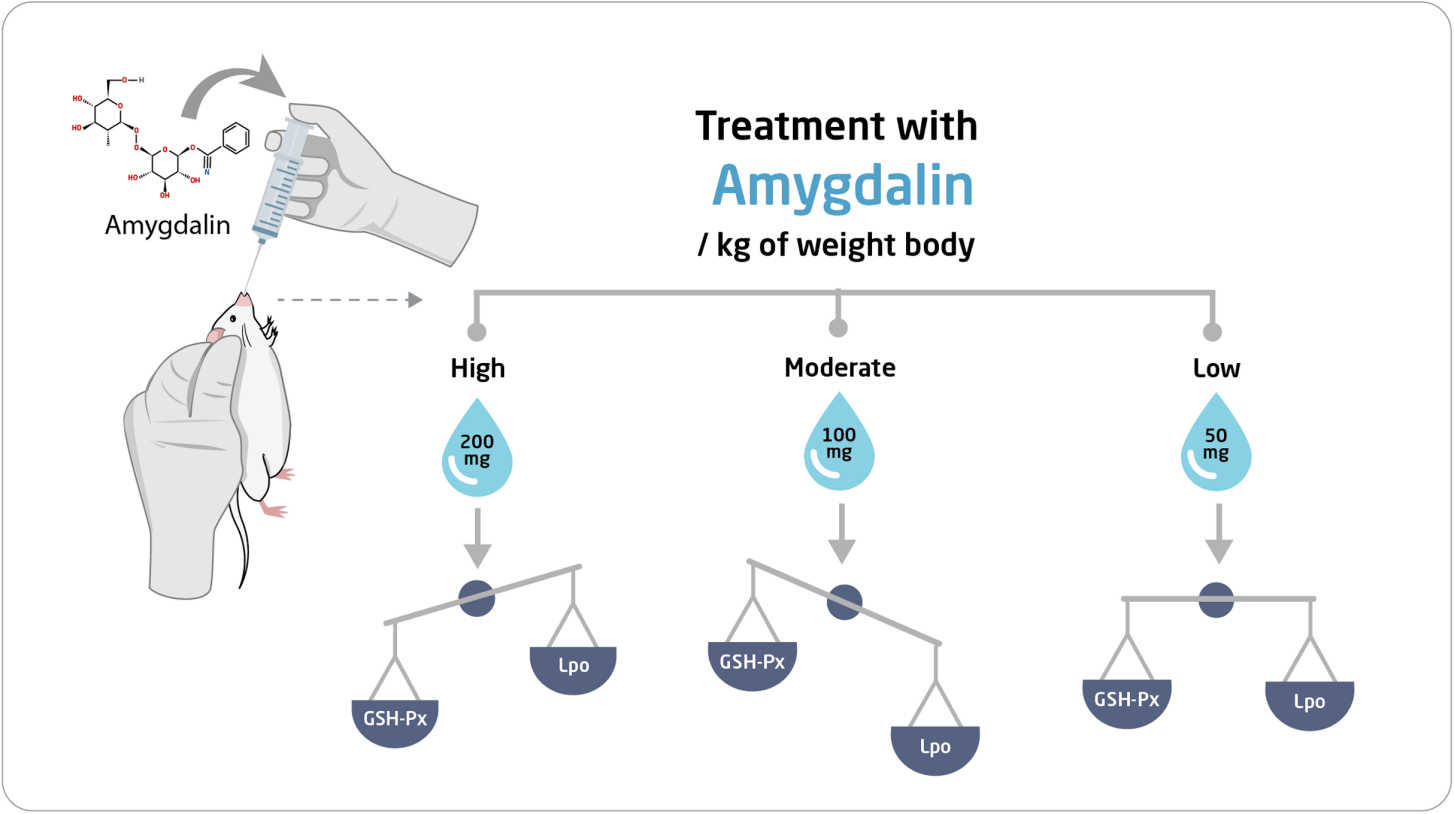
Schematic representation of the effect of amygdalin on experimental mice.

The results of this study (Fig. 8) revealed that low-dose amygdalin did not alter the mRNA expression of GSH-Px and SOD or the GSH-Px protein levels in the hepatic and testicular tissues. Moreover, low-dose amygdalin treatment did not result in a significant difference in the LPO levels in hepatic and testicular tissues, which is consistent with the findings of Hamada et al., who investigated the effect of *Eriobotrya japonica* seed extract, which has been shown to contain amygdalin, on oxidative stress and adriamycin-induced nephropathy in rats (Hamada et al., 2004). They found that the level of GSH in the renal tissue of rats significantly increased following treatment with the extract (7 mg/kg body weight), whereas the renal plasma and tissue levels of lipid peroxide significantly decreased. There were no significant differences in antioxidative enzymes in the renal tissues, suggesting that the effects caused by *E. japonica* seed extract occurred via direct antioxidative action (Hamada et al., 2004).

## Conclusions

A treatment of 100 mg/kg amygdalin showed the highest efficacy in upregulating GSH-Px and SOD mRNA expression. Furthermore, enhanced antioxidant enzyme activities in the studied tissues led to decreased LPO level, accompanied by mild histopathological features. However, amygdalin at a high dose exerted negative effects on the oxidative balance of hepatic and testicular tissues of mice. The conditions leading to toxicity should be further investigated to determine those suitable for the administration of safe and effective oral doses of amygdalin, as a potential new cancer therapy option in the future.

## Supplemental Information

10.7717/peerj.9232/supp-1 Supplemental Information 1 GSH-Px and SOD mRNA expression in the hepatic and testicular tissues of mice treated with three different doses of amygdalin (200, 100 and 50 mg/kg), as examined using sq-PCR.

## Additional Information and Declarations

### Data Availability

The following information was supplied regarding data availability: The raw measurements are available as a Supplemental File.
